# Approaching rehabilitation in patients with advanced glaucoma

**DOI:** 10.1038/s41433-022-02303-z

**Published:** 2022-12-16

**Authors:** Ashley D. Deemer, Judith E. Goldstein, Pradeep Y. Ramulu

**Affiliations:** 1 Lions Vision Research and Rehabilitation Center, Wilmer Eye Institute, Johns Hopkins School of Medicine, Baltimore, MD USA; 2 Dana Center for Preventative Ophthalmology; Glaucoma Division, Wilmer Eye Institute, Johns Hopkins School of Medicine, Baltimore, MD USA

## Abstract

Vision loss from advanced glaucoma is currently irreversible and impairs functional visual ability to effectively perform everyday tasks in a number of distinct functional domains. Vision rehabilitation strategies have been demonstrated to be effective in low vision populations and should be utilized in persons with advanced glaucoma to reduce disability and improve quality of life. Initial challenges to rehabilitation include an incomplete understanding of vision rehabilitation by the physician and patient, motivation to integrate rehabilitation into the plan of care, and availability of suitable providers to deliver this care. Physicians, working with well-trained vision rehabilitation providers can maximize function in important visual domains customized to the patient based on their needs, specific complaints, severity/pattern of visual damage, and comorbidities. Potential rehabilitative strategies to be considered for reading impairment include spectacle correction, visual assistive equipment, and sensory substitution, while potential strategies to facilitate driving in those deemed safe to do so include refractive correction, lens design, building confidence, restriction of driving to safer conditions, and avoiding situations where cognitive load is high. Mobility is frequently disrupted in advanced glaucoma, and can be addressed through careful distance refraction, behavior modification, home modification, mobility aids, walking assistance (i.e., sighted guide techniques), and smartphone/wearable technologies. Visual motor complaints are best addressed through optimization of lighting/contrast, sensory substitution, IADL training, and education. Special rehabilitative concerns may arise in children, where plans must be coordinated with schools, and working adults, where patients should be aware of their rights to accommodations to facilitate specific job tasks.

## Abstract (zh)

晚期青光眼导致的视力丧失是目前不可逆的, 对在许多不同功能领域有效执行日常任务的视觉功能有损伤的疾病。视力康复策略已被证明对低视力人群有效, 应该用于晚期青光眼患者, 以减少残疾和提高生活质量。康复的最初的挑战包括医生和患者对视力康复、将康复纳入护理计划的动机、以及是否有合适的人员提供护理理解得不充分。医生与训练有素的视觉康复医师合作, 可以根据患者的需求、具体的主诉、视觉损伤的严重程度/模式以及共患疾病, 最大限度地发挥患者在重要视觉领域的功能。阅读障碍的康复策略包括屈光矫正、视觉辅助设备和感官替代, 而提高安全驾驶的潜在策略包括屈光矫正、镜片设计、建立信心、限制在更安全的条件下驾驶, 避免认知负荷高的情况。晚期青光眼患者的行动能力经常受到干扰, 可以通过仔细的距离屈光、行为矫正、家庭矫正、行动辅助、步行辅助 (即视力引导技术) 和智能手机/可穿戴技术来解决。视觉运动方面障碍的主诉最好通过优化照明/对比度、感觉替代、IADL训练和教育进行解决。在儿童和已经工作的成人中可能会出现特别的康复问题, 因为儿童的康复计划必须与学校协调, 而对于已经工作的成人, 他们更应该意识到他们有权利为特定的工作任务而进行调整。

## Introduction

Vision loss from glaucoma is currently irreversible and leaves patients increasingly impaired in daily activities with worse disease, leading to less independence [1–3] and secondary health consequences such as falls and fractures [4–7]. Relatively little effort has been focused on the practical question of how to maximize visual ability (perform everyday, visually-mediated tasks) and minimize the effects of impairment in persons with glaucoma, particularly those with advanced disease. The goal of vision rehabilitation (VR) is to address this gap in care by focusing on strategies to improve individuals’ ability to perform everyday visually-mediated activities. Here, we focus on reviewing the types of functional difficulties experienced by persons with glaucoma, with an emphasis on how and when patients are affected, approaches to integrating rehabilitation into chronic care, and specifying rehabilitation strategies to improve quality of life.

### Incorporating rehabilitation into the model of ophthalmic care

Several medical disciplines have deeply incorporated rehabilitation into their model of care. Neurologists, for example, focus on the treatment and prevention of stroke, partnering with speech, occupational, and physical therapists to address the functional deficits resulting from the stroke. Similarly, orthopedic surgeons focus on surgical correction of bone/joint deficits, partnering with physical therapists to maximize post-surgical functional outcomes.

Within ophthalmology, significant barriers exist in the integration of rehabilitation into glaucoma care. First, unlike other specialties where there is often an acute event that triggers the need for rehabilitation, the chronic, gradual changes, and fluctuations in vision due to glaucoma obscure the right time to refer. Multiple attempts and conversations over time may be necessary to connect patients to rehabilitation. The glaucoma physician must continually consider disease progression along with change in function as they focus their discussions with patients over the course of the patients’ lifetimes. The physicians treating glaucoma must either take it upon themselves to guide patients through the rehabilitative process – a significant challenge given the limited time available during routine visits, the time-consuming process of understanding if and how function is impaired, and the lack of training in rehabilitation – or refer patients to a VR specialist. Referral to specialists may be limited by a lack of specialists (or known specialists) in the area, a limited understanding of what specialists do, and limited faith (physicians’ and/or patients’) that specialists can change patients’ lives.

### Vision rehabilitation can improve patient functionality

While few trials have specifically focused on the rehabilitation of glaucoma patients, several trials and observational studies have shown substantial improvements in visual ability with VR intervention in patients with vision loss from a mix of conditions. The effectiveness of low vision services has been assessed in numerous ways, including clinical performance measures (e.g., visual acuity (VA), reading speed) [8] patient-reported outcomes (e.g., questionnaires assessing the ability to perform every-day activities, changes in mood/depression) and surrogate measures (e.g., functional independence judged by therapists). The complexities of varied outcome measures make cross-study comparisons difficult [9]. Patient-reported outcomes using visual function questionnaires remain the predominant metric given the individualized nature of rehabilitation, and the importance of considering how each patient perceives his/her own functionality.

Strong evidence regarding VR effectiveness comes from the Low Vision Intervention Trial performed in the Department of Veterans Affairs Blind Rehabilitation Center, which studied an intensive 4–6 week inpatient program with coverage of visual assistive equipment [10]. In this randomized controlled clinical trial assessing patients with severe central vision loss (VA between 20/100 and 20/500 from macular disease), substantial improvements in self-reported visual ability were reported with VR as compared to the wait-list control group (Cohen effect size = 1.96, large effect sizes defined as >0.8) [10, 11]. In a multi-center US observational study examining usual care in private outpatient VR centers, where assistive equipment was not covered and mean visual acuity was slightly better (20/100), self-reported visual ability improved in nearly half of patients (47%), with smaller average effect sizes than the inpatient program, but still classified as large (Cohen effect size = 0.87) [12].

While not focusing specifically on glaucoma patients, these data prove that VR is effective and should be offered to patients interested in improving their visual ability. However, successful specialist referral requires that physicians provide patients with a convincing rationale for the benefits of rehabilitative services and set realistic expectations. As part of these conversations, patients are likely to have several questions which physicians should be facile in answering (Table 1).

**Table 1:** Frequent patient questions regarding vision rehabilitation (VR) with sample answers.

Question	Sample answers
What will they (VR specialists) do for me?	“They will help to maximize your remaining vision and recommend different strategies and ways to tackle everyday activities so that you may function as safely and independently as possible”.
Will I get new glasses?	“Evaluating the glasses prescription is a routine part of VR care and glasses will be prescribed if they improve your vision and you see benefit. However, glasses will not correct for the visual field loss from glaucoma” [20].
Am I going to keep seeing you?	“Yes, I will continue to follow-up with you to monitor the health of your eyes, while my colleague will help you live with your disease better, making sure you are as safe and functional as you possibly can be given your vision problems”.
I went before, but they didn’t help me.	“While no one can fully restore the vision or function lost from glaucoma, VR strategies involve adapting behaviors to make tasks easier. When you are ready and open, they may be helpful to you”.
I don’t have any problems, why do I need to see them?	“They can discuss strategies and demonstrate equipment that may help you in the future if you are not experiencing problems now”.
Is it covered by my insurance?	“Insurance does cover the costs of office visits to see a VR specialist for clinical care including rehabilitation training”. Coverage of visual assistive equipment, however, depends on the policies of the country in which you practice.
I already have a magnifier, what else are they going to do for me?	“There are many different types of visual assistive equipment, technology and resources that may be helpful for traveling, managing changes in lighting conditions and assisting with your daily activities inside the home and outside the home”.
So, does this mean I’m not going to get better?	“We are working together to maintain and maximize the vision you have now. Although we are not able to restore you vision back to normal at this time, the VR specialist can help you better use the vision you have”.

### Identifying referrals for vision rehabilitation

With a goal of improving visual ability, VR is best suited to patients reporting functional difficulties attributable to their vision, and whose vision is unlikely to meaningfully improve with further treatment. A challenge arises when handling glaucoma patients who describe no significant difficulties with visual ability at advanced stages of disease [13]. It is unclear if these individuals have capably adapted to their vision loss, have low demands, or do not want to share the true level of their disability. Practically speaking, it will be harder to convince persons with limited perceived functional difficulty to seek low vision care, though efforts to this end have potential value given that these individuals are at greater risk of harmful events such as falls, motor vehicle accidents, and future disability [4, 5, 14]. Thus, we would recommend at least introducing the idea of VR services in advanced disease, such that referral may be accepted by the patient over time. Variability in perceived function, based on individual patient preferences and visual demands, make establishing universal criteria about precisely when VR services should be offered difficult. A conservative approach is endorsed by the American Academy of Ophthalmology in their Vision Rehabilitation Preferred Practice Pattern and recommends discussing VR services when VA declines to worse than 20/40 in the better seeing eye [15]. When this acuity results from glaucoma, patients will invariably have severe visual field and contrast sensitivity damage; therefore a different threshold for discussing referral in all patients (i.e., a mean deviation worse than –15 dB in both eyes) may also be considered. As predicting the best timing for VR intervention and prognosis is difficult, a low threshold for education on and referral to VR services is likely good practice [16]. There is some evidence to suggest that patients report a decline in quality of life and visual function measures in even low levels of glaucotomatous damage. Notably, mild, unilateral loss has been shown to be associated with negative quality of life scores [17, 18] and newly diagnosed glaucoma patients with early damage also report loss of visual function [19]. As many clinical practices already include intake questionnaires for all new and some follow-up patients, this may be best screened by including a short, simple question addressing the patient’s functional status: “Are you having difficulty with any of your everyday activities because of your vision”?.

All demographic groups should be considered for VR services. Eligible patients may be of any age, including children who need assistance as part of their education (e.g., classroom learning, testing knowledge, interaction with other students, performing homework) to working-age adults who need support with occupational and family demands (e.g., job performance and accommodations, career aims, transportation, acquiring disability, etc.) to older, retired adults who may need assistance with everyday tasks facilitating independence (e.g., preparing meals, managing finances, housework) [1, 2]. Given the increasing prevalence of glaucoma with age and increased disease severity and greater comorbidity in older patients [20], many patients meriting referral will be older and may no longer be working as a result of their age and visual disability. Comorbid illness can be expected in many older patients with vision impairment, and should not inhibit referral for VR services.

Of note, prior VR services, or refusal to consider such services, should not be a contraindication for referring a patient to VR for care. It is to be expected that our patients’ visual demands will change with age, the waxing and waning of comorbid illness, and changes in their vision. As such, VR practitioners do best when they become life-long partners to optimize functionality. Similarly, patients not initially agreeable to seek such services may become so in the future as their life circumstances change.

One does not typically refer a patient to VR services, but often a patient-caregiver dyad or an entire family [21]. Most patients needing VR will require assistance with transportation and may need reinforcement of care recommendations [22, 23]. At times, there are emotional barriers to seeking rehabilitative services and the benefit of services can be reinforced by enlisting the support of friend and family accompanying patients to their visit, resulting in more successful referrals.

The system for providing VR care varies across health systems, with services provided by ophthalmologists, optometrists, occupational therapists, and others. Ultimately, a team approach is required in which the ongoing medical and VR care of the patient are integrated. Medical providers address the medical and surgical aspects of disease to protect against further visual impairment. However, most medical providers cannot also address the patient’s existing disability, which is better handled by VR specialists partnering in the care of the patient.

As detailed below, understanding the effects of the impairment on the patient and individualized patient preferences is time-consuming and requires clinicians with expertise in rehabilitation medicine and behavioral health. As such, these efforts are best undertaken by VR specialists with the time and expertise to focus on improving visual function and ability.

### Discussing and referring for rehabilitation

Several questions may arise when the suggestion of VR is broached with patients and their families. Examples of questions and sample answers are provided in Table 1. While the discussion of VR adds time to the visit, it is the standard of care when managing patients with non-correctable vision loss, and we argue here that it is time well spent. In many cases (i.e., advanced glaucoma patients with stable, low intraocular pressures after surgery), VR is the therapy most likely to add value to their lives. The critical first step in educating and informing patients about the utility of VR services lies with the referring practitioner; those who doubt the utility of VR services are likely to devalue and/or deferral VR care.

Patients place tremendous trust in their treating physicians (this is especially true for advanced glaucoma patients, many of whom have required surgical treatment) and therefore the suggestion for rehabilitation is best received from treating physicians. Such recommendations should not be seen as a relinquishment of care, but rather a new partnership leading to optimal care of the patient: “I have a colleague who helps me provide the best possible care for patients with functional difficulties due to glaucoma. I will continue to treat and monitor your disease, ensuring that your glaucoma is under control and that you do not lose any further vision. My colleague will help you live with your disease better, making sure you are as safe and functional as you possibly can be given your vision problems.” While such discussion should commence at the initiation of treatment of advanced glaucoma, given the time limitations involved in routine visits, it is reasonable that an initial physician recommendation for VR services be saved for a visit where the patient is in a stable condition, leaving more time for the discussion of VR as an important portion of care.

Patients often focus on restorative therapy and hope that a change in eyeglasses, medication or surgery will “fix” their vision. As this is less likely and may result in the disappointment, setting expectations for the VR visit and persuading patients to obtain an evaluation as part of their total eyecare is critical to success and engagement in the process. It is helpful to let patients know that although refractive improvement may offer some improvement in vision [24], effects may be limited in the setting of severe visual field and contrast sensitivity deficits. Rather, patients should plan on describing the specific daily activities that are important and difficult to perform. These activities can be organized into functional domains for the purposes of developing plans of care. We describe the functional domains: reading, driving, visual information/general “seeing”, mobility, and visual motor activities. Below, typical problems experienced by glaucoma patients in these domains are described, along with approaches to rehabilitating these problems.

## Functional domains

### Reading

Reading is the most common chief complaint in patients seeking VR services irrespective of ocular diagnosis [25] and among the best predictors of patient-reported visual ability and quality of life [26, 27]. Fortunately, in glaucoma, sentence-level reading ability remains relatively intact until advanced disease stages when central vision becomes impaired [28]. However, several aspects of reading can be disturbed in glaucoma even with preservation of central vision, including difficulty with sustained reading and reading comprehension [29]. These difficulties may reflect that, in advanced glaucoma, reading requires a greater degree of mental focus [30]. Various reading functions are performed in varied conditions (e.g., reading caloric content on supermarket package, reading a menu in a dim restaurant, social media posts on smartphone, etc.). While spot reading of high contrast material may be possible in many advanced glaucoma patients, complaints more often manifest in activities that require sustained reading or reading under poor lighting [31], where worse performance has been noted [32].

When evaluating and managing reading in glaucoma, it is important to determine whether the etiology of the deficit is from (1) loss in the paracentral visual field – causing scotomatous-type loss, (2) loss in visual acuity, (3) loss in contrast sensitivity, or a combination of factors [33]. In patients accessing VR services, contrast sensitivity moderately correlates with visual acuity *(r* = *−0.52)* [34]. However, in almost one-fifth (18%) of patients, VA is mildly impaired while contrast sensitivity is severely reduced [35]. Patient-reported reading concerns in the presence of intact near visual acuity and fluent continuous reading should direct the practitioner’s attention to loss in contrast sensitivity. In these cases, measuring contrast sensitivity with Pelli-Robson or Mars charts can confirm whether treatment options should focus more on lighting and manipulating print electronically with a tablet or smart phone, rather than magnification.

Although there is not one ideal measure of reading ability, the testing method should match the type of reading the patient wishes to perform (e.g., spot reading, continuous reading). One common reading test is MNRead [36], a chart which measures reading at 18 print sizes ranging from a Snellen equivalent of 20/400 to 20/6.3 (M size 8.0 to 0.13). MNRead can be very useful in assessing reading speed, which becomes especially important when VA is unaffected but the visual span (the number of characters that can be recognized at a single glance) is affected due to loss in paracentral vision [37, 38]. Anecdotally, some advanced glaucoma patients will read slower at larger print sizes and faster with smaller print due to a spared central area. In these cases, simply measuring threshold visual acuity is unlikely to manifest the deficit. In addition to near threshold VA and reading speed, MNRead provides a measure of critical print size (smallest size print which near maximum reading rate is achieved). MNRead metrics referenced to normally-sighted children and adults can be helpful when determining print size accommodations for individualized education plans or educating patients about optimal font size when reading [39].

A subset of patients with glaucoma report difficulties with reading endurance. These patients report that they can only read for a period of time before their reading vision blurs, their eyes “tire” or words start “jumping”. Inadequate functional reserve in these cases may be due to relative loss in retinal sensitivity in or near fixation affecting scanpaths. Dry eye does not appear to be the primary reason for fatigue in these patients given evidence for similar degrees of corneal staining before and after reading (Ramulu, unpublished). Dry eye is, however, a common comorbidity in this population and therefore dry eye therapy may be indicated [40]. Worsening corneal parameters have been noted after sustained reading in dry eye patients, and may play a role in reading fatigue in some glaucoma patients [41, 42]. When reading endurance is a concern, testing patients with longer passages can help elicit the problem [31].

For most patients, reading ability is dictated by function in the better-seeing eye and therefore it is common for reading to be adversely affected when there is loss in the eye with better VA, contrast sensitivity or visual field (paracentral or central) [43]. Paracentral scotomas can affect both reading speed and accuracy [44, 45]. While evidence on the relationship between reading speed and scotoma location (e.g., to the left or right of fixation) is not definitive, it is essential to diagnose such loss (absolute or relative) such that rehabilitation strategies and rehabilitation potential coincide with findings. As magnification is one of the most common rehabilitation treatment options, care must be taken to avoid magnifying the effects of scotomas; contrast enhancement must instead be maximized to minimize loss in sensitivity. When absolute scotomas impact reading speed at all print sizes, patients should be educated that optical and electronic solutions are unlikely to be satisfactory to regain normal reading speed, and sensory substitution (i.e., audio books, podcasts) is the primary treatment modality.

#### Reading rehabilitation strategies

VR management of reading deficits in glaucoma can be categorized into: (1) conventional approaches such as spectacle correction, (2) enhancement with visual assistive equipment and (3) use of non-visual approaches. Table 2 outlines treatment considerations in response to common patient-reported reading concerns.

**Table 2:** Reading concerns and treatment considerations.

Common patient reported concerns	Treatment considerations
• Print too small • Words run together • Difficulty keeping place when reading from line to line	• Spot/task lighting (e.g., flashlight) • Large print hard copy • Enlarging print size on electronic devices (i.e., tablets, computer) • Optical hand and stand magnifiers • Electronic magnification or closed-circuit television systems (CCTV) • Conversion of text to speech
• Print is not dark enough • Unable to read in poor lighting conditions (e.g., menus at a restaurant)	• Task lighting with LED, natural spectrum lighting, sunlight • Reverse polarity settings (white letters on black background)
• Larger print more difficult to read • Fatigue when reading	• Use of a line guide • Minify print to maximize field and minimize scanning • Conversion of text to speech

Spectacle correction and lighting changes can help when there is uncorrected refractive error and contrast sensitivity is near normal. A higher add power (e.g., +4.00) may be considered if visual field loss is not present near fixation, in which case higher adds often adversely affect reading speed function. Trial framing and testing accuracy and speed using the intended reading approach (e.g., newspaper or mobile phone) is important to assess before prescribing as is assessing the impact of rivalry from the poorer seeing eye. Additionally, emphasizing to patients that multiple strategies may be needed to address different types of reading demands (i.e., phone, mail, book, etc.) can help set expectations. When field loss is close to fixation, reducing the multifocal addition power to increase the working distance and adding external visual assistive equipment may be needed. Considering a single vision lens design rather than a multifocal in order to maximize the area of accessible reading power can be helpful when the working distance is sustained (e.g., computer); patients may have the inconvenience of switching spectacles when a different viewing distance is needed.

Even with best spectacle correction, enhancing perceived contrast using task lighting and/or electronic presentation is critical. Modifying lighting in patients with glaucoma is often necessary when hard copy demands it, such as reading item labels or signing documents, and when continuous text is a rehabilitation goal. While previous studies of reading ability have generally been done in clinical settings with good lighting [29, 32, 38], in-home lighting levels are generally much worse, with an average home lighting of less than 300 lux for most rooms other than the kitchen with lights on. Worse lighting has significant effects on visual ability, with one study finding nearly half of advanced glaucoma patients reading two or more lines worse on visual acuity testing at home as opposed to in the clinic [46]. Testing and prescribing different lighting types (e.g., LED, natural spectrum, incandescent) and brightness levels is essential and can be one of the most effective treatment options when visual field loss is close to fixation. Additionally, educating patients on the prolonged time needed to visually adjust to task lighting can minimize rejection of this helpful solution. Positioning of lighting such that the light source is close to the reading material often provides the most benefit with early paracentral vision loss. Additionally, portable lighting should be considered, as smart phone and traditional flashlights can be invaluable to assist with spot reading. Of note, in end-stages glaucoma, patients may report that task lighting actually “washes out” their reading vision. In these cases, spectacle correction has a more limited effect and additional contrast enhancement with electronic devices may be needed (further details below).

Maximizing reading ability and optimal spectacle correction should also consider the effects of the poorer seeing/less functional eye when it appears to be impacting overall reading ability. Ocular or binocular rivalry occurs when the better eye sees worse binocularly in the setting of a poorly-seeing worse eye; with poorer reading function may suppress naturally or the patient may close or cover the eye. While evidence suggests this phenomenon is indeed present in conditions such as macular degeneration [47], one study did not find better-eye contrast sensitivity to differ from binocular contrast sensitivity in the context of a poorly-seeing worse eye [48]. Nevertheless, when patients complain of difficulty resulting from a worse-seeing eye making it difficult to see out of the better-seeing eye, the refractive evaluation and near assessment should consider how to minimize distracting input from the poorer-seeing eye. Anecdotally, reading ability in patients with rivalry sometimes is better when VA is not maximized in both eyes (e.g., modified worse-eye add power), or artificial occlusion is prescribed (e.g., Bangerter filter).

##### Visual assistive equipment

**Fig. 1:**
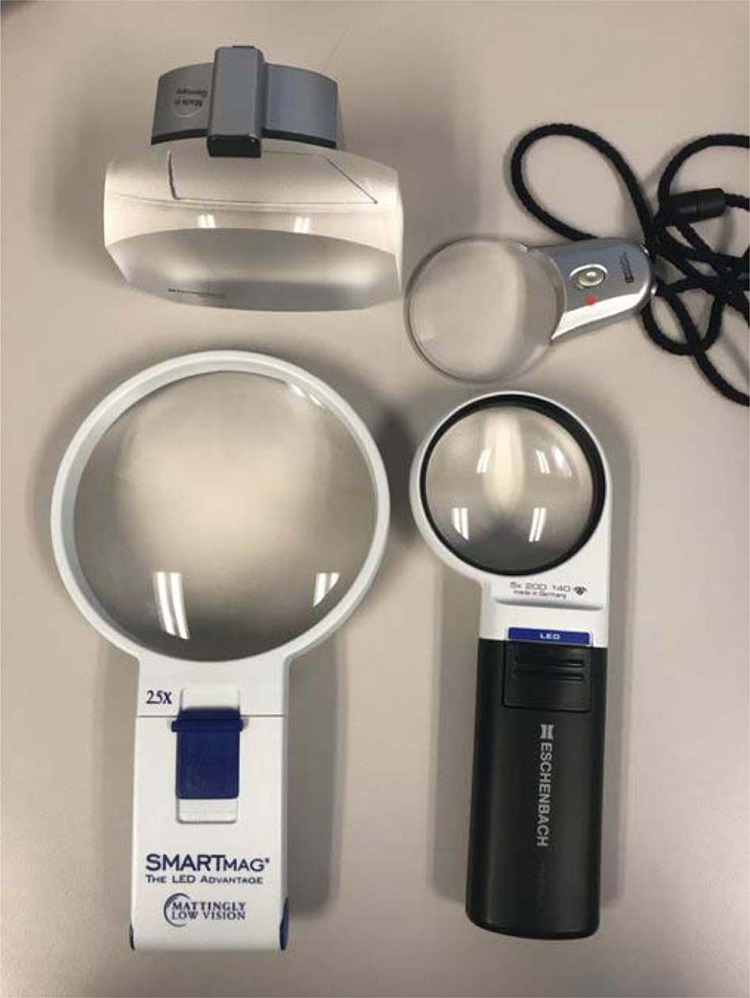
Examples of illuminated optical stand and hand magnifiers. Optical stand is shown in upper left, along with three hand magnifiers.

**Fig. 2:**
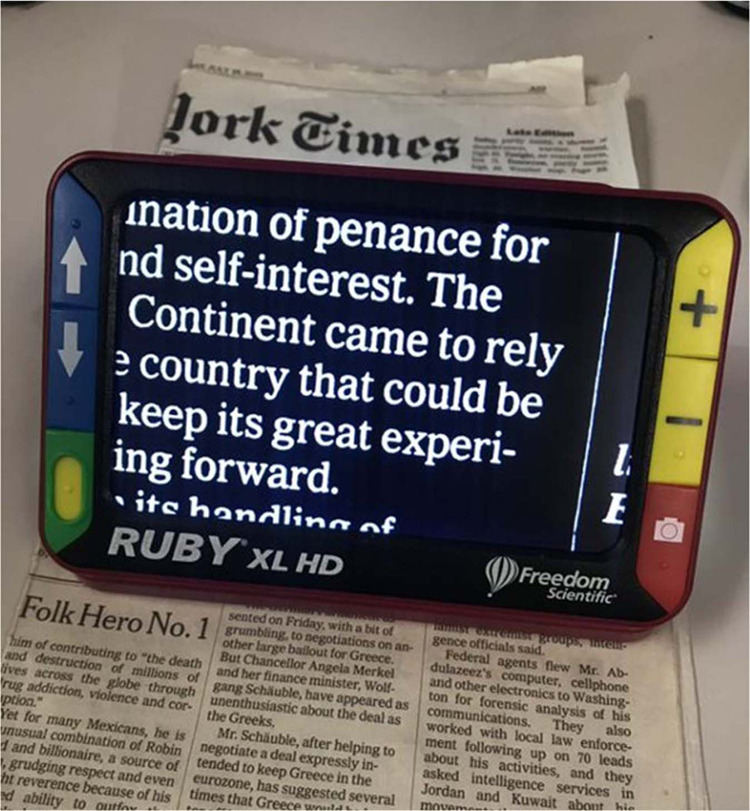
Example of reverse polarity contrast enhancement with a portable closed-circuit television (CCTV). The ability of a commercially available portable CCTV to convert normal polarity text (black letters on white background) to reverse polarity (white text on a black background) is shown.

Magnification systems coupled with controlled lighting may be very helpful to patients with reading deficits due to VA and contrast sensitivity loss [49]. Empirically, low optical magnification (6–8 diopters) with a hand or stand magnifier coupled with illumination can often facilitate effective spot reading (Fig. 1). Electronic smart phone magnification is convenient to magnify text with an app, or simply by using the camera zoom. Phones and tablets offer flexibility in controlling the brightness, size and color of the text, and background, making rehabilitation easily customized to the patient’s unique and variable reading demands. These “over-the counter” consumer tools, when set up properly (spacing, margins, short-cuts, etc.), can be effective in enhancing reading function, with reading (particularly spot reading) being the most common condition in which one smartphone video magnifier app was used [50]. When delivering VR care, we have found glaucoma patients commonly prefer white text on black background (referred to as reverse polarity, Fig. 2) while controlling the brightness of the white text with settings or wearable tinted filters. This enhanced contrast and magnification can also be obtained through stand-alone Closed-Circuit Television (CCTV) systems of varying size and portability.

##### Sensory substitution

Obtaining assistance from other people, using smart home devices, listening to audio books, using Optical Character Recognition (OCR) technology, and reading braille are examples of strategies available when vision enhancement is not satisfactory to achieve patient-directed reading goals. OCR technology has vastly improved in the past decade, with free apps available on smart phones and incorporation into wearable technology. OCR refers to the electronic conversion of images of typed or printed test into machine-encoded text which then is converted to speech output. This typically is facilitated by software overlaid on camera technology. Although the advancement of this technology has improved access to reading material, it is not a panacea. OCR does not make reading charts, graphs, legends, or names easy, and navigating using OCR takes training, skill, and patience to master. For advanced glaucoma patients who identify reading as an important goal, and in whom text enhancement is not a solution, focused sensory substitution strategies become an essential strategy. Difficulty with this approach often resides in convincing the patient to “read” using a new strategy. While some may resist acceptance of this approach, it may be best practice to continue with follow patients to assess readiness for behavioral change. The psychology literature describes the stages of change as multifaceted and dynamic [51, 52], and patients must sometimes move from repetitive education on rehabilitation options to smaller, less-intrusive implementation strategies before transitioning into full sensory substitution (e.g., use of a talking watch or voice commands to read smarphone messages).

### Driving and transportation

Patients presenting for VR services commonly report driving-related concerns as transportation is typically necessary for independent daily life, and public transportation systems are often inadequate for older adults with vision loss [25]. However, the variability in impairment from glaucoma and the response to changes in vision (e.g., transportation provided by others, modification of driving practices, lack of acceptance or change in driving practices) may result in some glaucoma patients compensating well, while others voice concerns. Patients may hesitate to share driving-related concerns with their doctors for fear of losing privileges, challenging the management of driving in glaucoma. Critical aspects in managing driving/transportation include determining if patients are fit to drive per local standards, guiding patients about their fitness to drive, specifying adaptations to driving in appropriate individuals, and aiding alternative transportation options for those who cannot drive safely.

Observational studies of on-the-road performance show patients with glaucoma are more likely to have worse on-road driving performance marked by deficits with lane maintenance, scanning, speed, and planning ahead [53, 54]. Also crash risk per mile generally increases with the presence and severity of glaucoma [55–57]. Translating these observations from studies to the individual is difficult as the connection between the impairment and response is so variable. Common patient-reported concerns and treatment considerations regarding driving are listed in Table 3.

**Table 3:** Driving and transportation concerns and treatment considerations.

Common patient reported concerns	Treatment considerations
• Difficulty with reading road signs until close	• Refraction and prescribing best correction (careful consideration of fellow eye (if rivalrous) • Single vision distance glasses if dashboard clarity is not a concern • Global Positioning System (GPS) use • Limiting driving to familiar areas • Telescope use in the form of a bioptic
• Difficulty viewing dashboard/GPS	• Consider need for lower add power in “driving” vs. “reading” bifocals • Speedometer smartphone app
• Trouble in bright sun	• Wrap-around/form fitting tinted spectacles with side shields • Medium to dark tinted lenses (with or without polarization) • Limiting driving to certain lighting/weather conditions • Use of sun visor and hat
• Trouble at twilight, night, entering tunnels/garages and oncoming headlights	• Light yellow wrap-around tinted spectacles with side shields • Alternative transportation options/discontinuation of driving under these conditions
• Judging depth/distance	• Practice, training and reinforcement with CDRS^a^ • Maximization of refractive correction with consideration of rivalry • Car safety/alert features
• Uncertainty regarding driving ability/skills	• Car safety/alert features • Recommendation re: minimization of driving frequency, distance, and ultimate cessation • Behind the wheel evaluation with CDRS^a^ • Education on alternative transportation options (county services, ride share programs)

^a^Certified driving rehabilitation specialist.

Driving-related rehabilitation management typically involves: (1) maximizing acuity, (2) addressing variability in lighting and glare, (3) reducing the cognitive load of driving, and (4) building confidence in driving ability. Although loss in peripheral visual field and contrast sensitivity contributes significantly to driving difficulty, currently field expansion options are limited in their effectiveness, often leading patients to use larger eye/head scanning strategies. Improving eye and head scanning strategies are sometimes intuitive as patients lose additional peripheral or central vision gradually allowing for slow adaptation; other times training and feedback behind the wheel may be necessary. However, the utility of these scanning mechanisms is unclear, as excessive scanning may increase the awareness of peripheral threats, but reduce awareness of the scene immediately in front of the driver [58]. Tinted lenses to modify light transmission, though may be ineffective when lighting is variable.

For public safety, it is important for glaucoma specialists to ask patients if they are currently driving. Asking all patients with moderate to severe visual field damage annually, or even every visit, will ensure that higher-risk cases are not overlooked. Questions can be simple, such as “Do you ever drive?” and “Do you ever drive at night?” Patient responses provide the specialist with enough information to consider whether further evaluation (i.e., education, VR referral) may be needed while not significantly adding to the visit time. Regular inquiries also keeps consideration of future driving cessation on the patient’s mind and may minimize surprise and defensive postures when suggestions about modifying driving behavior are raised. In cases where continued driving is questionable, or there may be opportunity for rehabilitation to improve driving ability (e.g., refraction, glare control, etc.), referring the patient for a VR evaluation, where further assessment regarding driving capacity can be performed by a different specialist, is often helpful. As these conversations can be emotional, difficult, and lengthy, it may be helpful for these discussions and counseling sessions to occur separate from the medical management. Conversations are often best had in the company of family, who can help support proper decision-making that might be unpleasant for the patient.

#### Driving and transportation rehabilitation strategies

##### Maximizing acuity

Problems with timely road-sign recognition and other distance detail are best addressed by addressing uncorrected refractive error. Specifically, trial frame refraction allows the clinician to observe how the patient functions in “free-space” and detect meaningful refractive change, ocular rivalry (does the patient close an eye when instructed to view binocularly?), eccentric viewing (does the patient turn their head or their eyes?), or a positive response to distance magnification, in which case a telescope might be indicated to improve spotting acuity during driving. Depending on the findings, changing the refractive correction, lens design (progressive to single vision only), or less commonly incorporating a telescope may prove beneficial, with ideal therapy guided by patient preference and practitioner experience.

##### Changes in lighting

Variability due to time of day, year, and weather conditions can create uncertainty among patients with glaucoma [59], and many glaucoma patients restrict their driving practices in response [35, 60]. When changes in lighting (i.e., tunnels, wooded areas) impair driving performance, trialing and prescribing different filters/tints may improve speed of response and comfort in managing changing luminance behind the wheel for some. Several driving conditions are expected to be more difficult for glaucoma patients, and discussions about driving for counseling the patient about (1) limiting night driving appropriately, (2) maintaining confidence by staying in familiar areas or modifying routes that offer more controlled conditions, and (3) using restriction as a bridge to eventual driving cessation, which allows time for adaptation and adjustment, and may minimize the risk of depression.

##### Cognitive load

Effective driving involves adequate cognitive ability including divided attention skills and quick reaction times. While not studied specifically for driving, reduced ability to divide attention has been associated with functional outcomes in glaucoma [30]. Even without cognitive impairment, the increase in concentration and attention required in the context of vision loss can cause anxiety and significant fatigue. Patients often report difficulty driving at the end of a workday and VR efforts should address work-related accommodations (telework and modification of drive times where possible).

##### Building confidence

Loss in vision often is associated with loss in driving confidence. When there is physician or patient uncertainty regarding fitness to drive, the gold standard is a behind-the-wheel driving skills evaluation with a Certified Driving Rehabilitation Instructor. Generally, instructors are certified to perform evaluations and provide training for novice and experienced drivers with vision impairment. Reporting requirements on releasing results of the driving assessment to the Department of Motor Vehicles vary by state/region so it is important to be aware of specific local regulations and inform the patient accordingly. These evaluations can be very helpful to assess fitness as well as to recommend behavior modifications to allow for continued driving. For example, being trained to implement head and eye scanning strategies, use of tinted lenses, and incorporating narrative driving can improve skill to the point that driving is more effective and may continue. Ongoing conversations with patients about ride share options, cars with advanced safety systems, and relying on others who are driving may improve confidence or support adaptation while maximizing patient and public safety.

### Visual information

As part of any rehabilitation evaluation, patients are queried about subjective clarity during distance viewing as this affects the ability to read signage, navigate in unfamiliar territory and recognize faces. Gathering of distance visual information can be thought of as “seeing” and is dependent on both visual acuity (VA) and visual field, and is especially important for social engagement. Similar to other eye conditions, patients with vision loss from glaucoma may report ongoing decline in their vision/visual information despite what appears to be stable VA and visual field. This can challenge the determination of definite disease progression (and the need for more aggressive intervention) vs. visual fluctuations, which are very common in persons with severe glaucoma.

VR evaluations can be helpful in deciphering differences between disease progression and symptomatic fluctuation as face-to-face time permits lengthy discussion of the patients’ observations of their vision, assessing VA, contrast sensitivity, refraction, and examining paracentral loss in sensitivity. When VA, contrast sensitivity and visual field are unchanged from prior measures, patients can be reassured that observed changes are more likely to represent fluctuation. In some patients, a change in refraction may recover VA to baseline levels – again reassuring the patient [24]. Alternatively, evaluations may reveal stable VA and visual field, but a decline in contrast sensitivity, thus offering an explanation to support patient symptoms. Most patients with glaucoma maintain relatively good acuity until advanced stages of the disease and therefore may observe a disconnect between noticing subtle changes in their vision and being told their vision measures the “same” or is “stable”.

#### Visual information rehabilitation strategies

An objective and subjective benefit to a change in distance refractive correction is likely one of the most meaningful treatments to improve “seeing” and therefore the refractive evaluation is a key component to the rehabilitation assessment. Proper refractions of persons with advanced glaucoma require different techniques that may not be easily performed by technicians, or even most practitioners. For example, careful measurement of VA (lights on, lights off, use of eccentric viewing, reversing polarity on VA projection chart) helps set the conditions for trial frame refraction. A phoropter is not preferable if there is central vision loss, as it creates an artificial viewing environment, limiting the available light, making eccentric viewing difficult, and restricting easy demonstration of larger magnitude lens changes. Common patient observations and treatment considerations regarding visual information are detailed in Table 4.

**Table 4:** Visual information concerns and treatment considerations.

Common patient reported concerns	Treatment considerations
• Unable to see the entire scene/missing components of the scene • “Not enough light”	• Increase distance from the scene until central and peripheral visual field is maximized • Education on systematic scanning • Add lighting • Increase brightness and/or contrast levels in environment, electronic screens • Use of sensory substitution (examination of shape, gait, voice, facial recognition technology)
• Vision not clear/difficulties seeing details/facial expression	• Trial frame refraction • Evaluate for uncorrected cylinder or axis change due to trabeculectomy, tube surgery or conjunctival scarring • Evaluate binocular visual function; maximizing clarity in both eyes vs. minimizing correction in an eye that is rivalrous • Get closer to objects or people where possible • Monocular or binocular telescope use • Tinted filters
• Eyes don’t work together	• Evaluate for anisometropia or misalignment with or without diplopia • Consider risks/benefit to enhancing binocularity with spectacle, contact lenses or prism (Fresnel or ground-in) vs. monocular occlusion
• Glasses don’t help enough • Glasses are not right	• Consider single vision or contact lens instead of multifocal • Education of expectations of distance correction • Severe contrast sensitivity loss will typically minimize subjective benefits to objective high contrast VA improvement • Education on the limitation of light transmission through spectacle lenses, minimizing the value of VA improvement

Visual field is critical to gathering and processing visual information during visual searching. Although most visual search problems manifest as deficits in visual motor and mobility domains, disorganized saccadic activities can affect the perception of distance clarity and object recognition. When visual field loss occurs near fixation but VA is preserved, counseling and educating patients with visual information problems can be helpful, even if restoration is not possible.

### Mobility

Mobility is a primary complaint amongst patients with glaucoma [18], as vision loss due to glaucoma predisposes patients to activity limitations, adverse events (i.e., falls) [4, 6], psychological consequences (i.e., fear of falling) [61–63], and reductions in physical activity [64, 65] that can lead to secondary health concerns. Below, various strategies for appropriately balancing physical activity and movement while minimizing falls are discussed. Table 5 also outlines the common mobility complaints of patients and offers strategies to combat negative mobility effects from visual field, visual acuity, and contrast sensitivity loss.

**Table 5:** Mobility concerns and treatment considerations.

Common patient reported concerns	Treatment considerations
• Bumping into things or people • Missing curbs and steps	• Reduce speed when walking • Walk with someone for assistance • Use of mobility aid i.e., long white cane for obstacle detection and identification • Limiting travel to familiar areas • Remove all clutter from floors • Add bright markers to curb or step edges • Reverse telescope/minifier
• Unable to read signs • Difficulty seeing details	• Maximize distance refraction • Consider single vision distance correction to avoid bifocal line interference • Monocular or binocular telescope
• Difficulty in dim lighting environments • Difficulty transitioning from varying lighting conditions	• Flashlight or add additional lighting • Tinted filters
• Weakness/poor physical strength • Dizziness or balance • problems	• Non-slip mats • White or standard support cane or walker • Grab-bars and use of hand railings • Physical therapy • ENT evaluation/vestibular rehabilitation

#### Mobility rehabilitation strategies

##### Maximizing distance clarity

As with most functional domains, the first step in rehabilitating mobility involves maximizing distance clarity with a careful distance refraction. Lens design may be important as well. While evidence is inconclusive regarding the type of lens best suited for reduced vision visual field loss [66–69]. it may be helpful to consider single vision opposed to multifocal lens designs in glaucoma. It is common to observe individuals with advanced glaucoma become more hesitant and look down when ambulating, and recent evidence suggests longer delays before initiating walking with more severe glaucoma damage, particularly in changing light conditions [70]. With a multifocal lens, looking down at the floor without proper tilting of the head to maintain a view through the optical center of the lens will cause blurred vision at distance, suggesting that patients would be forced to choose between unusual head tilt angles and blurring of floor terrain/hazards. Education on moving the head and gaze down to maintain this alignment with the optical center, or switching to a single vision lens design, can provide better optical clarity and may improve mobility. Of note, switching to a single vision lens design may not be a good option for those who prioritize the convenience of having one pair of glasses for varying optical distances, or who experience difficulty finding objects.

##### Behavior modification

Given the slowly progressive nature of glaucoma, affected individuals tend to naturally adapt over time. To improve accuracy and safety, patients are encouraged to slow their walking speed [71]. Patients may make other adaptations as well, such as adapting a wide-based gait [72]. Glaucoma patients more frequently bump into objects [73], and encouraging scanning for obstacles and orientation and mobility training focusing on maximizing conceptual knowledge and cognitive processing like utilizing memory can aide in navigation [74, 75]. Patients should be advised to wear appropriate shoes that fit well, have a firm heel to provide stability, and have a textured sole to prevent slipping. It is also good habit to have patients hold onto hand railing when available. In addition, adapting an active lifestyle early in the disease process should be advised to maintain muscle strength needed to compensate for vision impairment in advanced disease stages [76], and a more active lifestyle may protect against further glaucoma damage [77].

##### Home modifications

Most falls in glaucoma patients occur inside the home [78, 79] and trends are likely to increase with travel and social distancing restrictions. It is therefore important to address the home environment to minimize falls, especially as the number of home hazards are not less in persons with more advanced glaucoma [79]. Maintaining a clutter-free, well organized space is a pragmatic approach to avoid tripping over unseen objects. Having sturdy, well-built furniture is critical if needing to grab on to something when balance is lost. It may be helpful to avoid low contrast items like glass furniture and add bright contrast makers like tape to furniture and/or steps [80, 81]. Table 6 outlines a number of other home modifications considerations by room. Referral to a low vision occupational therapist is often recommended to evaluate and implement the changes needed in the home [82], and particular emphasis should be given to rooms where most falls occur in patients (stairs and bedroom most commonly, followed by the bathroom and living room) [78]. As vision loss is often progressive, it may be prudent to discuss future planning i.e., moving into a single level home. Early adaptations are beneficial as many patients state that the more familiar they are with their home the less problems they experience.

**Table 6:** Home modifications by room/area (centers for disease control and prevention).

Home environment	Modification considerations
Bathroom	• Shower or bath chairs
	• Hand bars/grab bars
	• Non-slip rubber mats or textured strips on floor of the tub or shower.
	• High contrast markings (e.g., white tub and dark rubber mat)
	• Shampoo and conditioner - use a rubber band to mark and distinguish the bottles
	• Organize and declutter the bathroom so it is easier to find everyday items
	• Toothbrush - white bristles and colored toothpaste or squeeze toothpaste into mouth
	• Colored bath towels on a white wall for higher contrast
	• Black toilet seat over a white toilet for higher contrast
	• Good diffuse and task lighting throughout the environment
Floors	• Remove rugs or tape down edges of rug
	• High contrast rugs - light floors with darker colored rugs; dark floors with lighter color rugs
	• Eliminate floor clutter and keep objects off the floor
	• Tape down cords and wires
Kitchen	• Re-arrange items to avoid excess pivoting, bending down, and turning around
	• Put commonly used items within easy reach around waist level
	• Never stand on a chair to reach items; consider a step-stool or ask someone else for assistance
	• Add task lighting and/or under cabinet lighting
	• High contrast outlets and light switches
	• Curtains and blinds maybe necessary in the home to block out the extra lights if too much glare
	• Label spices with bolder labels/label maker
Stairs and steps	• Stair climber
	• Necessities on a single level in the home
	• Add handrails to both sides of stairs
	• Repair any loose stairs and make sure carpet is firmly attached
	• Non-slip treads on steps
	• Light switches on the top and bottom of the steps
	• Bright overhead lighting
	• Mark the top two and bottom two steps with high contrast tape to mark the beginning and end of the step
Bedroom	• Add bright light bulbs
	• Curtains and blinds maybe necessary if too much glare
	• Place lamps close to bed within easy reach; avoid dark lamp shades
	• Install night lights or motion sensing lights for an easier to see path when dark
	• Create a clear walking path from bedroom to/from bathroom
	• Place mobility aid accessible to bed for use during the night trips to bathroom
Entry way	• Place shoes in a designated area by the front door; avoid placement in the middle of the room
	• Place purse and keys in a designated location off floor
	• Remove rugs or tape down edges of rug
	• Overhead lighting

For patients living alone at risk for falling, it is advisable to discuss the use of a medical alert system or personal emergency response system. These systems consist of a wearable device which is connected to a central station. If the user experiences a fall and they are unable to assist themselves in getting help, a wearable button can be pushed to connect the user to the system operator who can help assess the problem and dispatch emergency services to their location.

##### Mobility aids

Other compensatory strategies to improve mobility in advanced glaucoma includes non-optical strategies. For example, a long white cane is traditionally used to compensate for poor peripheral visual field or significantly impaired visual acuity, enabling obstacle detection and avoidance [82, 83]. With training, it provides tactile information about the environment, and serves as a marker to the public that the user has a visual impairment. Many glaucoma patients are hesitant to take up white cane use because: 1) they can persist with behavior modifications, such as slowing down, and 2) there is no single event or moment where the individual experiences a drastic enough change in vision to overcome the stigma of using of a white cane. While many patients are comfortable and safe with simple behavioral adaptions, white cane use is recommended for persons considered a high fall risk who desire to travel independently outside the home and have adequate physical ability.

**Fig. 3:**
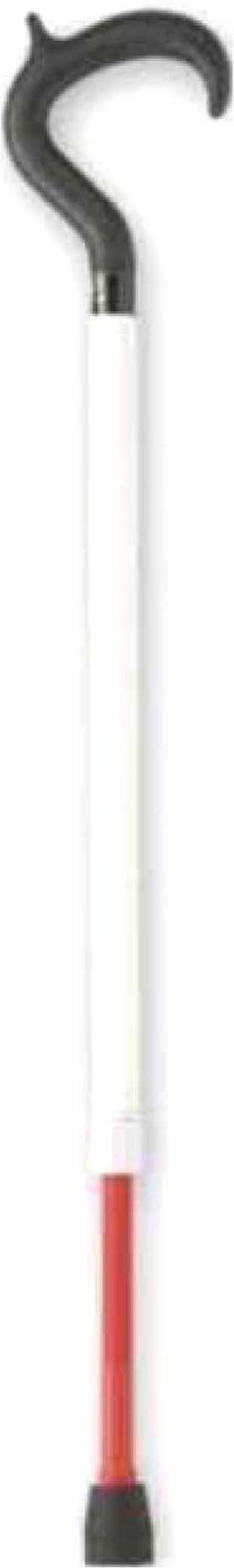
White and red support cane commonly used by visually impaired individuals with comorbid physical limitations. Support cane has the advantages of previewing the environment ahead, and offering physical support to prevent stumbles or falls.

A long white cane requires a patient has sufficient physical strength and balance to ambulate. As risk factors for glaucoma increase with age, it is often the case that many patients have co-morbid loss affecting their physical strength, balance, and endurance [84]. In these cases, a long white cane may not be adequate to provide the needed support to enable safe, independent mobility. Recommending physical therapy and strength training can be helpful in combination with use of supportive devices like a support cane. Specifically, a white/red support cane (Fig. 3) can allow patient stability while also identifying that a person has vision loss. Similar to the long white cane, the support cane can be used to “poke around” for surface preview as long as the patient has enough stability to do so, though the support cane was not specifically designed for surface preview and may not be sufficient for complete independent mobility. Use of the support cane would, however, be preferred over “toe searching” and may be superior to no mobility aid.

In contrast with white cane use, the use of a guide dog is aimed at obstacle avoidance, rather than detection. Extensive training is needed and guide dogs are only considered when individuals are proficient white cane users who still reports disability and have the cognitive and physical ability to co-navigate with a dog.

##### Assistance from others

Utilizing the assistance from another individual is another method used by patients with advanced glaucoma. A common adaptation is the sighted guide technique, in which a sighted person (friend, family member, or caregiver) guides the patient both physically and verbally. When educating patients and their guides on this technique, the visually impaired individual is advised to hold on to the sighted guide’s elbow or shoulder to more naturally feel body movements as they are navigating through space. Ample verbal instruction is also needed to properly warn and advise the patient of upcoming turns and narrow spaces.

##### Advances in technology

Useful smartphone apps and wearable technology have advanced rehabilitation treatment options for severe glaucoma patients. Using a portable camera, glaucoma patients can connect to a sighted individual, typically crowd-sourced volunteers or trained customer service agents. These sighted individuals see what the user sees through the camera and provide guidance and feedback to assist in orientation. While this connection made through technology can help in several functional domains, it is specifically helpful for mobility. A skilled cane user, for example, may be able to independently navigate in the neighborhood by visual memory and good cane skills, but can experience difficulty if there is road construction unexpectedly extending to the sidewalk. By connecting to a sighted agent via a smartphone app and camera, the agent can then utilize the information from the live video connection as well as the smartphone’s global positioning system (GPS) to survey the situation and guide the user on an alternate route.

### Visual motor and other instrumental activities of daily living (IADLs)

Visual motor tasks integrate visual skills with motor skills. Loss of visual perception information such as depth cues may reduce hand-eye coordination and efficiency with tasks like reaching and grabbing. As glaucoma is a bilateral, yet typically asymmetric, disease [85, 86] it is common to see visual field deficits occur and/or progress at different rates in each eye. When one eye has significantly more visual field damage than the fellow eye, or has deficits which affect fixation, patients often shift to functioning monocularly. Even if the less affected eye is still highly functional on its own, the loss of binocularity can cause problems with visual ability, particular visual motor activities that involve depth perception [87]. Difficulties are more likely seen when there is sudden, asymmetric vision loss for which there is insufficient time for individuals to adapt. A loss of depth perception can affect activities such as participation in sports, hobbies like crafting, creating artwork, handiwork, and other inside and outside the home IADLs that involve reaching, grabbing, pouring, cooking, and cleaning [88].

#### Visual motor rehabilitation strategies

Limited solutions exist for problems that arise from loss of binocularity and depth perception. Most all rehabilitation strategies are non-optical and require adaptation by the patient. These strategies include 1) adjusting lighting, 2) incorporating sensory substitution, 3) IADL training, and 4) patient and family education, as outlined Tables 6 and 7.

**Table 7:** Visual motor concerns and treatment considerations.

Common patient reported concerns	Treatment considerations
• Difficulty seeing details	• Add task lighting to directly illuminate stove top, counters, etc.
• Finding objects	• Use of high contrast items – black and white cutting boards, plates, placemats, etc.
• Knocking items over	• Education on scanning the scene
• Misjudging distances	• Emphasize organization, consistent placement of items and a clutter free environment
	• Use of non-visual or tactile skills
	• Reduce speed to improve accuracy

##### Lighting and contrast

As with many tasks, lighting is extremely important for optimizing contrast when performing various IADLs involving visual motor function [89]. Task lighting in all areas around the home assist in enhancing detail. For example, additional lighting above the stove or over counter tops is helpful during preparing meals [90]. Flashlights or other portable lights should also be considered. Various colored household items can be modified to maximize contrast. For example, when cutting a white onion, a black cutting board can provide better background contrast.

##### Sensory substitution

**Fig. 4:**
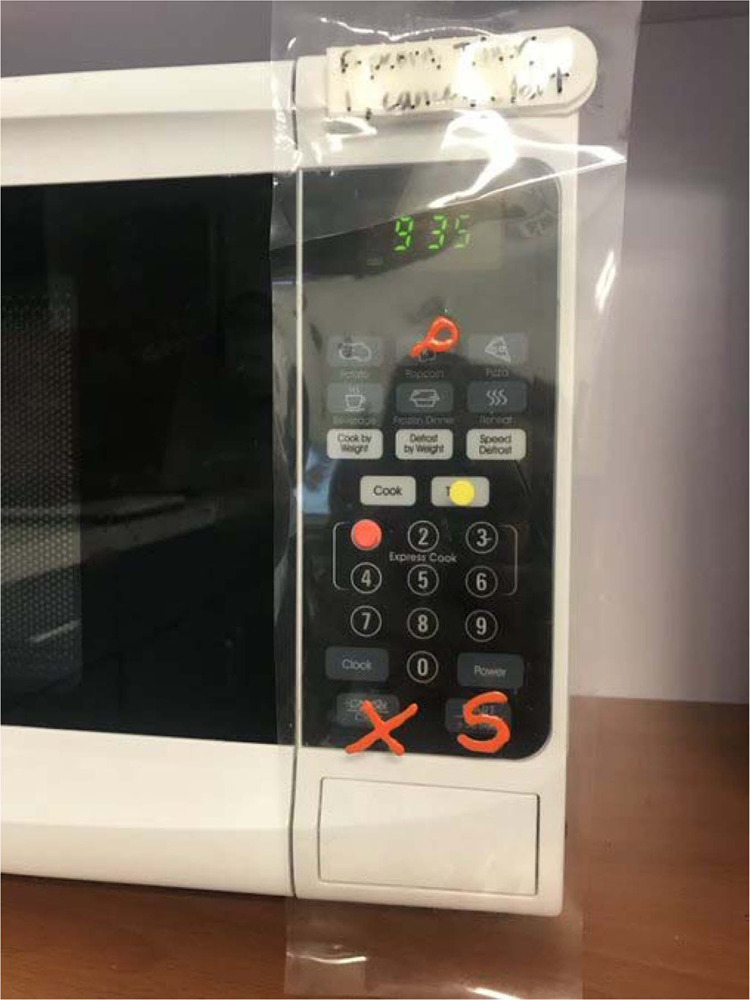
Example of tactile marking on microwave. Tactile markings, in this example, allow for recognition of critical buttons allowing the microwave to be used.

When vision is not sufficient to safely perform visual motor tasks, tactile and auditory information can be used to assist. For example, adding brightly colored, textured bump dots to appliances (Fig. 4) and using one’s fingers for tactile information when cutting and pouring [90] are useful ways to rely on the sense of touch rather than vision. When pouring liquids, patients can use their finger to feel the liquid level as it reaches the top of the cup, and touching the bottle to the cup can be helpful in preventing spills. With hot liquids, a device called a hot liquid level indicator will produce an auditory alarm when the liquid nears the top of the cup.

##### IADL training

Outpatient and in-home occupational therapy (OT) services are utilized to identify problems with activities of daily living, to provide patients with solutions to these problems, and to practice these strategies. Often, home safety modifications such as avoiding clutter and maintaining organization are emphasized. OT providers can also practice activities like cooking, cleaning, and laundry directly in the home, utilizing therapist-defined strategies based on input from the patient [91]. If a patient is no longer safe at home, it is also prudent to discuss alternative options. These may include eliciting the support from family members, friends/neighbors, or other healthcare facility professionals in the patient’s home or moving into a new living environment with more support.

##### Education

Even when solutions are limited, education on how glaucoma affects function can be meaningful in the patient rehabilitation. Educating the patient on how monocular function may impair visual motor and IADL functions can assist in preventing ongoing frustration by helping patients realize their difficulty is visual, and not because of cognitive decline, age, or other deficiencies. Understanding the reasons behind impairment can also encourage the patient to continue practicing new adaptive strategies. A good book to reference, written from the patient perspective, is titled *A Singular View* by Frank Brady [92] Available in large print and audio, the book provides valuable insight into the challenges of monocular functioning and lends support to individuals and their family members who may be experiencing similar difficulties.

### Special considerations for children and adults in the workplace

While age is a significant risk factor for glaucoma, there are still a number of individuals affected in childhood and in the younger, working-age population [93, 94]. When rehabilitating these patients, additional considerations should be kept in mind.

Congenital glaucoma is a chronic condition requiring rehabilitation throughout life. As individuals go through certain milestones defined by age and changes in vision, rehabilitation strategies will often need to be adapted. As such, it is important that these patients (and their families) develop long-term partnerships with VR specialists. In particular, it is important to ensure patients receive appropriate accommodations throughout their educational career. Often a formal agreement, referred to in the U.S. as an Individualized Education Plan (IEP), between the school and the student and his/her family will need to be established to protect the patient’s access to accommodations and services. These accommodations are aimed at giving equal access to education and to prepare the visually impaired student for the workforce.

When considering VR in the working-aged adult, it is important for VR providers to understand the job requirements and associated visual demands. Many job duties involve the aforementioned functional domains, so it is important to critically analyze specific job tasks and functions in order to utilize the appropriate strategies detailed above. For each important job tasks, a strategy or accommodation should be identified and discussed. If there is no visual or sensory solution to perform tasks independently, other adaptive strategies should be incorporated, including outsourcing certain tasks and/or use of a personal assistant.

Fatigue is another important factor to consider as many jobs require a heavy visual demand, and a 40-hour work week can be difficult for in those with advanced glaucoma. For example, while visual acuity may be relatively unimpaired, contrast loss, scotomas and/or visual field loss can significantly lower reading speed over time [29], necessitating greater time for long passages [31], and visual fatigue throughout the day. For those working, it is important to consider other accommodations when visual demand is greater than what the patient may be able to accommodate.

It is also important to discuss the rights patients have in the workplace. In the United States, the Americans with Disabilities Act of 1990 (ADA) is a federal law that prohibits discrimination on the basic of disability, including vision impairment. Title I of the ADA prohibits qualified individuals with disabilities to be discriminated against in the job application procedures, hiring, firing, advancement, compensation, job training, and other terms, conditions and privileges of employment. Under this title, *reasonable accommodations* are defined as a change or adjustment that would allow individuals equal access to applying for a job, performing job functions, and enjoying access to benefits available in the workplace. These regulations will vary by nation, though many nations prohibit discrimination on the basis of disability, and require employers to provide reasonable accommodations to disabled workers. Some examples of reasonable accommodations include computer accessibility hardware and software, live readers and other devices to access text auditorily, and time off for someone who needs treatment. If accommodations cannot be made, a VR provider may counsel on options for applying for disability benefits.

## Conclusion

We have done our best here to introduce the reader to a variety of methods to help glaucoma patients function better in their daily lives. We hope the reader has taken away a few general messages from the article.

### Summary

#### What was known before

- Glaucoma patients, particularly those with advanced disease, experience a broad range of functional difficulties.
- Traditional aspects of ophthalmic care, which focus on diagnosis, detection of progression, treatment to prevent progression, and visual improvement by addressing issues such cataract and refractive error, remain important but do not address many underlying daily problems.

#### What this study adds

- The reasons for functional difficulty with a given task are complex, and often depend on details of when, where, for what purpose, and for how long various tasks are performed.
- Solutions for addressing functional difficulties are equally, if not more, complex and need to be tailored to the reasons for difficulty and tested to make sure that they are right for the patient.
- Many suggestions for how to improve functionality are derived from the expertise of practitioners that work routinely with visually impaired patients and have not been explicitly proven by research. Indeed, formal study of how best to rehabilitate specific functional impairments in glaucoma is an important area for future investigations.
- It is unrealistic to expect the glaucoma doctor, even after reading and digesting this article, to be able to professionally counsel patients on how to improve their function. Rather, it is most important they identify others in their community (VR practitioners, occupational therapists) with whom to partner, and learn how best to communicate to patients the importance of incorporating VR into their care plan.
- How to incorporate VR into care may differ greatly in different systems of medicine, and an important area of future research is to document the types of difficulties experiences with advanced visual field damage in developing world settings, and developing the infrastructure for providing rehabilitative care in areas where VR specialists may not currently exist.
- The ultimate roles of the glaucoma provider are to serve as educator and advocate to educate patients about the benefits of VR care, to advocate bringing in and embracing colleagues who can provide rehabilitative care to patients with advanced disease, and to advocate that rehabilitative services (and the equipment that is often prescribed) be provided to patients who may benefit.
